# Does statin suppress oxaliplatin-induced peripheral neuropathy in patients with colorectal cancer? A single-center observational study

**DOI:** 10.1007/s00520-023-08134-2

**Published:** 2023-10-28

**Authors:** Kazuaki Okamoto, Hiroaki Nozawa, Shigenobu Emoto, Koji Murono, Kazuhito Sasaki, Soichiro Ishihara

**Affiliations:** Department of Surgical Oncology, The University of Tokyo, 7-3-1, Hongo, Bunkyo-ku, Tokyo, 113-8655 Japan

**Keywords:** Colorectal cancer, Statins, Neuropathy, Oxaliplatin, Adjuvant chemotherapy, CAPOX

## Abstract

**Background:**

Oxaliplatin-induced peripheral neuropathy (OIPN) is a common and dose-limiting toxicity that markedly limits the use of oxaliplatin and affects quality of life. Statins have been shown to exert neuroprotective effects in preclinical settings. The aim of the present study was to clarify whether statins prevented OIPN in patients with colorectal cancer (CRC) receiving adjuvant CAPOX therapy.

**Methods:**

We examined 224 patients who received adjuvant CAPOX therapy for CRC between July 2010 and December 2021 at our hospital. Patients were divided into “Statin” and “Non-statin” groups based on statin use. Details on and the adverse events of adjuvant CAPOX therapy were examined in association with statin use.

**Results:**

Thirty-one patients (14%) were treated with statins. There were no intergroup differences in the relative dose intensity or number of CAPOX cycles between the Statin and Non-statin groups. In total, 94% of patients in the Statin group and 95% of those in the Non-statin group developed OIPN (*p*=0.67). The severity of OIPN was similar between the two groups (*p*=0.89). The frequency of treatment delays in CAPOX did not significantly differ between the Statin and Non-statin groups (16% vs. 11%, *p*=0.45).

**Conclusions:**

The efficacy of statins to attenuate OIPN during adjuvant CAPOX therapy was not apparent in the current study. Further studies are needed to confirm the present results.

## Introduction

Colorectal cancer (CRC) is the third most deadly and fourth most commonly diagnosed cancer worldwide [1, 2]. Previous randomized clinical trials (RCTs) revealed that 5-fluorouracil (5-FU) effectively prevented the recurrence of CRC after radical resection [3–5]. In another series of RCTs, better survival was achieved by oxaliplatin in combination with 5-FU than by 5-FU alone for stage II and III colon cancer [6–8]. Therefore, a growing number of locally advanced CRC patients have received oxaliplatin-based adjuvant chemotherapy [9–11].

Major adverse events of oxaliplatin include peripheral neuropathy, myelosuppression, and gastrointestinal reactions, such as diarrhea and stomatitis [12, 13]. These unfavorable effects of oxaliplatin may disrupt the treatment plan and reduce the drug compliance of CRC patients. Oxaliplatin-induced peripheral neuropathy (OIPN) leads to drug reductions or discontinuation and impairs the quality of life of patients [14, 15]. Despite intense preclinical and clinical research, no drugs have been recommended to prevent the development of OIPN [16].

Animal studies previously demonstrated that statins, HMG-CoA reductase inhibitors, exerted neuroprotective effects by attenuating oxidative stress [17, 18]. However, clinical studies reported conflicting findings; a RCT showed that rosuvastatin ameliorated diabetic polyneuropathy [19], whereas a prospective cohort study revealed that reductions in cholesterol levels increased the rate of painful neuropathic syndromes [20]. Therefore, the efficacy of statins to prevent neuropathy in humans remains unclear.

Regarding the relationship between statins and OIPN, only one retrospective study reported that the incidence of OIPN decreased with statin use in patients with several cancer types [21]. Therefore, we herein investigated whether statins prevented OIPN in CRC patients receiving capecitabine and oxaliplatin (CAPOX) therapy.

## Materials and methods

### Patients

We investigated consecutive Japanese patients who underwent radical surgery for primary CRC and received adjuvant CAPOX therapy between July 2010 and December 2021 at the University of Tokyo Hospital. Patients who received preoperative chemoradiotherapy without oxaliplatin were included. We excluded patients who had previously received duloxetine, a medication recommended for the treatment of OIPN [9, 10].

The present retrospective study was approved by the Ethics Committee of the University of Tokyo (No. 3252-[16]).

### Adjuvant CAPOX therapy

At our hospital, we recommend adjuvant chemotherapy to CRC patients based on the latest guidelines of the Japanese Society for Cancer of the Colon and Rectum [22]. However, chemotherapy regimens are modified at the physician’s discretion according to the patients’ age, performance status (PS), and other comorbidities. CAPOX therapy consisted of the intravenous infusion of 130 mg/m^2^ oxaliplatin and the oral administration of capecitabine at a dose of 1,000 mg/m^2^ twice daily for two weeks. The treatment course was repeated every three weeks [23].

### Data extraction

We retrieved the following data from our prospective database and patient medical charts: age, sex, body mass index, Eastern Cooperative Oncology Group PS, comorbidities, such as diabetes mellitus, cardiac, pulmonary, renal, and hepatic diseases, statin use, the primary location, the pathological classification of tumors according to the American Joint Committee on Cancer staging manual [24], the relative dose intensities of chemotherapeutic drugs, the number of CAPOX cycles, dose reductions, unscheduled treatment delays, and adverse events graded according to Common Terminology Criteria for Adverse Events version 5.0 [25].

We divided patients into two groups according to statin use: the ‘Statin’ and “Non-statin” groups.

### Statistical analysis

Statistical analyses were performed using JMP Pro 16.2.0 (SAS Institute, Cary, NC, USA). All variables were summarized as medians (ranges), means ± standard deviations, or numbers (percentages). Quantitative variables were compared using the Mann-Whitney *U* test. Qualitative variables were compared using Fisher’s exact test or the chi-squared test with Yates’ correction where appropriate. All reported *p*-values were two-sided, and results were considered to be significant when *p*-values were <0.05.

## Results

**Fig. 1:**
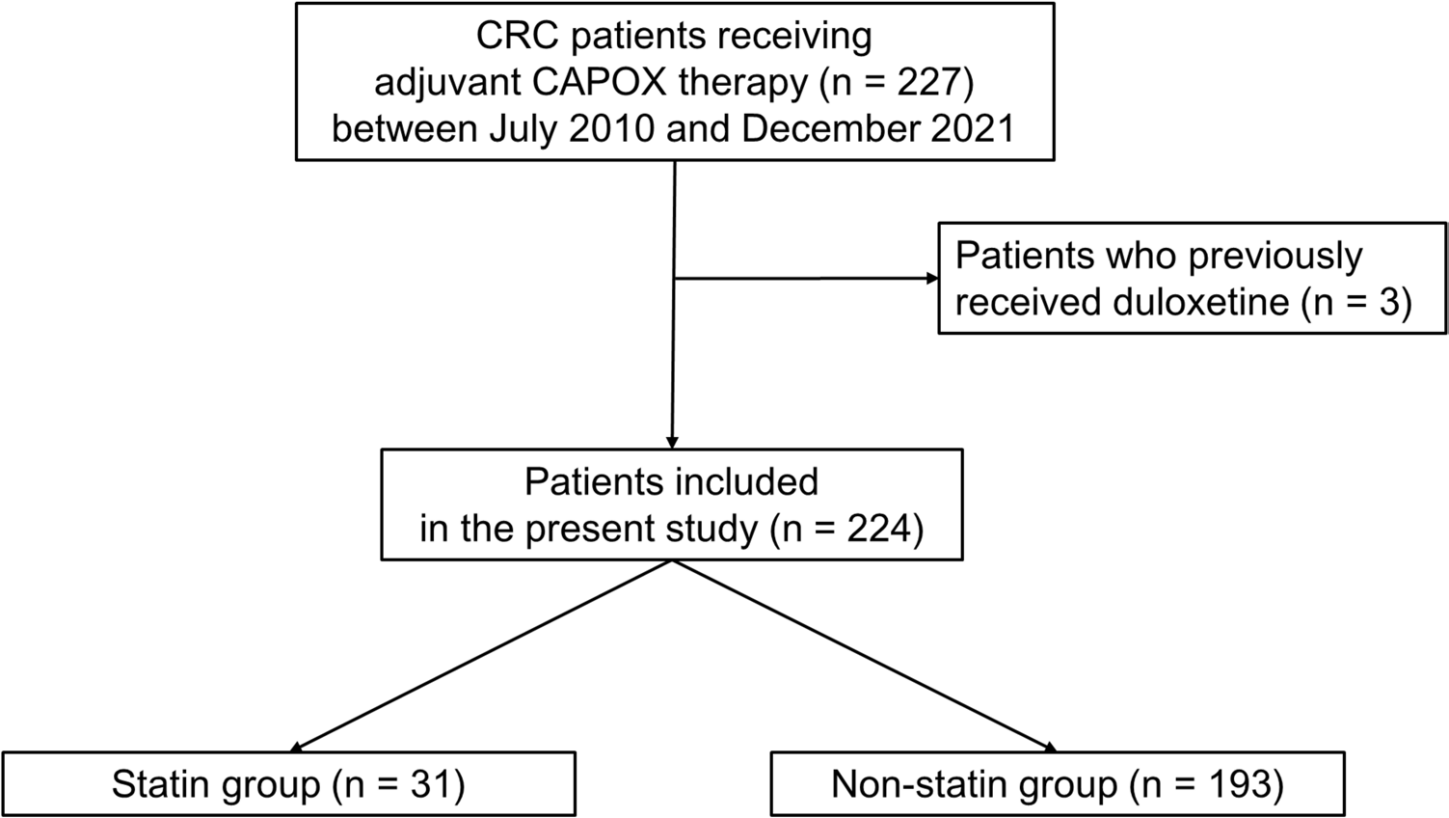
Study flow diagram for analyses

A total of 224 patients who received CAPOX therapy were included in the present study. Thirty-one patients (14%) received statins during adjuvant CAPOX therapy (Fig. 1). Statins used in the Statin group are shown in Table 1.

**Table 1:** Used statins in Statin group (*n*=31)

Names of statins	Number of patients (%)
Rosuvastatin	11 (35%)
Atorvastatin	7 (23%)
Pitavastatin	7 (23%)
Pravastatin	5 (16%)
Fluvastatin	1 (3%)

Values are presented as the number of patients (%)

Table 2 summarizes the characteristics of patients according to statin use. Patients in the Statin group were older than those in the Non-statin group (69 vs. 56 years old, *p*<0.001). Body mass index was higher in the Statin group than in the Non-statin group (*p*=0.023). In addition, the Statin group included more patients with diabetes mellitus, hypertension, cardiovascular disease, and dyslipidemia than the Non-statin group (*p*=0.008, *p*=0.019, *p*<0.001, and *p*<0.001, respectively). No significant differences were observed in other parameters between the two groups.

**Table 2:** Clinicopathological parameters of patients according to statin use

Variable	Statin group (*n* = 31)	Non-statin group (*n* = 193)	*p*-value
Demographic data			
Age, years	69 (43-79)	56 (26-82)	<0.001
Sex, male	15 (48%)	114 (59%)	0.26
Body mass index, kg/m^2^	23 (19-34)	22 (15-39)	0.023
ECOG PS			0.26
0	30 (97%)	192 (99%)	
1	1 (3%)	1 (1%)	
Comorbidity			
Diabetes mellitus	9 (29%)	22 (11%)	0.008
Diabetic neuropathy	0 (0%)	1 (1%)	1.0
Hypertension	13 (42%)	43 (22%)	0.019
Cardiovascular disease	7 (23%)	8 (4%)	<0.001
Pulmonary disease	5 (16%)	13 (7%)	0.074
Renal disease	0 (0%)	1 (1%)	1.00
Hepatic disease	0 (0%)	3 (2%)	1.00
Dyslipidemia	21 (68%)	7 (4%)	<0.001
Primary tumor location			0.73
Colon	16 (52%)	106 (55%)	
Rectum	15 (48%)	87 (45%)	
Pathological stage			0.11
I	1 (3%)	4 (2%)	
II	0 (0%)	24 (12%)	
III	27 (87%)	142 (74%)	
IV	3 (10%)	23 (12%)	
Preoperative chemoradiotherapy	5 (16%)	25 (13%)	0.58

Values are presented as the number of patients (%) or median (range)

*ECOG PS* Eastern Cooperative Oncology Group Performance Status

The treatment details of adjuvant CAPOX therapy are reviewed in Table 3. There were no significant differences in the relative dose intensity or number of cycles between the Statin and Non-statin groups.

**Table 3:** Treatment details of CAPOX according to statin use

Variable	Statin group (*n* = 31)	Non-statin group (*n* = 193)	*p*-value
RDI			
Capecitabine, %	83.8 ± 22.9	84.6 ± 17.0	0.65
Oxaliplatin, %	84.7 ± 19.9	85.6 ± 18.7	0.83
Number of chemotherapy cycles	7 (1-8)	8 (1-8)	0.19
Completion of eight cycles	15 (48%)	120 (62%)	0.15

Values are presented as the number of patients (%), median (range), or mean ± standard deviation

*RDI* relative dose intensity

Table 4 shows comparative adverse events during CAPOX according to statin use. In total, 94% of patients in the Statin group and 95% in the Non-statin group exhibited OIPN of any grade (*p*=0.67); both groups showed a similar distribution of the severity of OIPN (*p*=0.89). Moreover, grade ≥2 OIPN often occurred as early as at the end of the second cycle of CAPOX regardless of statin use (*p*=0.99). The incidence of dose reductions or treatment delays in CAPOX due to OIPN did not significantly differ between the Statin and Non-statin groups (13% vs. 12%, *p*=1.0 and 16% vs. 11%, *p*=0.45, respectively). The overall incidence of grade 3/4 adverse events other than OIPN was 23% in the Statin group and 30% in the Non-statin group (*p*=0.43).

**Table 4:** Details of adverse events, including OIPN, during CAPOX according to statin use

Variable	Statin group (*n* = 31)	Non-statin group (*n* = 193)	*p*-value
OIPN, all grades	29 (94%)	183 (95%)	0.67
Severity			0.89
Grade 1	7 (23%)	50 (26%)	
Grade 2	20 (65%)	122 (63%)	
Grade 3	2 (6%)	11 (6%)	
Onset of grade ≥2 OIPN (number of chemotherapy cycles)	2 (1-7)	2 (1-8)	0.99
Dose reduction due to OIPN	4 (13%)	24 (12%)	1.0
Treatment delay due to OIPN	5 (16%)	22 (11%)	0.45
Grade 3/4 adverse events, except OIPN	7 (23%)	57 (30%)	0.43
Leukopenia	0 (0%)	5 (3%)	1.0
Neutropenia	2 (6%)	27 (14%)	0.39
Thrombocytopenia	1 (3%)	7 (4%)	1.0
Anorexia	3 (10%)	7 (4%)	0.15
Nausea/Vomiting	1 (3%)	4 (2%)	0.53
Diarrhea	1 (3%)	5 (3%)	0.60
Fatigue	1 (3%)	3 (2%)	0.45
Allergy	0 (0%)	2 (1%)	1.0
Hand-foot syndrome	1 (3%)	3 (2%)	0.45
Liver dysfunction	0 (0%)	5 (3%)	1.0

Values are presented as the number of patients (%), median (range), or mean ± standard deviation

## Discussion

Although OIPN is a major dose-limiting adverse event of oxaliplatin, there are currently few preventive or treatment measures. Animal models of chemotherapy-induced peripheral neuropathy, including OIPN, have been established since the late 2000s [26–28], and many drugs have been examined as potential medications [29]. Preclinical studies previously suggested the neuroprotective effects of statins [17, 18, 30, 31]. However, only one study investigated the relationship between statins and OIPN in clinical settings, and subjects (277 patients) received different dose intensities of oxaliplatin with various drug combinations [21]. To the best of our knowledge, this is the first study to investigate the efficacy by which statins prevent OIPN during CAPOX therapy in CRC patients. In our cohort of 224 patients, we did not detect any relationships between statin use and the incidence or severity of OIPN.

Regarding the mechanisms of OIPN, previous studies suggested that OIPN is caused by ion channel dysfunction, glial activation, nuclear DNA damage, mitochondrial damage, neuroinflammation, and oxidative stress [16, 32]. Among these factors, statins were shown to have the potential to attenuate oxidative damage; two research groups reported that statins exerted neuroprotective effects through the suppression of superoxide formation in preclinical models [30, 31]. In 2022, a new study indicated that statins prevented OPIN in a rat model by inducing *Gstm1* mRNA, which promotes the detoxification of reactive oxygen species [21].

Despite promising findings from preclinical studies, clinical evidence suggested that the administration of statins to patients was associated with the development of neuropathy [33, 34]. The reasons for these discrepancies remain unclear; however, a narrative review on statins and neuropathic pain reported that reductions in low-density lipoproteins by statins may prevent the delivery of vitamin E, an essential factor for supporting healthy neural tissues [35]. A deficiency in vitamin E may disrupt nerve fibers, fat metabolism, and mitochondrial transport, which collectively result in increased neuropathic pain [35]. Further studies are needed to elucidate the complex relationship between statins and neuropathy.

Previous studies reported that OIPN occurred in 80–95% patients after treatment with oxaliplatin [36–38]. Similarly, in our Japanese cohort, 94% of patients receiving statins and 94% without statins developed OIPN. However, in the study by Zamami et al., only 65% of Japanese statin users developed OIPN during oxaliplatin-based therapy [21]. These inconsistent findings may be attributed to differences in patient backgrounds; we only included CRC patients, while Zamami et al. examined patients with various cancer types [21]. Although treatment details were not reported in that study, the intensity or dosing frequency of oxaliplatin may have differed between their study and ours.

The relationship between OIPN and diabetes mellitus currently remains unclear; however, a few previous studies suggested that patients with diabetes mellitus were more likely to develop early-onset and persistent OIPN than non-diabetic patients [39–41]. In the present study, the Statin group comprised more patients with diabetes mellitus than the Non-statin group (29% vs. 11%). This imbalance may have contributed to the similar incidence and severity of OIPN between the two groups, even if statins protect against neuropathy.

There are several limitations that need to be addressed. This was a retrospective study conducted at a single hospital. In addition, the number of patients receiving statins was relatively small, which may have caused type II errors. Besides diabetes mellitus, the intergroup disparities in age, body mass index, hypertension, cardiovascular disease and dyslipidemia may have contributed to the similar incidence and severity of OIPN between the two groups, even if statins protect against neuropathy. Moreover, the present study may have included a selection bias; adjuvant chemotherapy may not have been selected for patients who already had neuropathy and patients with dyslipidemia who were likely to have severe concurrent comorbidities. We also included some patients with a short follow-up, which may hamper the evaluation of long-term changes in chronic OIPN. Furthermore, the duration of and adherence to statin therapy were not examined.

## Conclusions

In CAPOX therapy for CRC patients, the effectiveness of statins for reducing the incidence or severity of OIPN was not observed in our cohort. The frequency of dose reductions or treatment delays was also independent of statin use. Further studies with a larger patient cohort are needed to confirm the present results.

## Data Availability

The datasets during and/or analyzed during the current study available from the corresponding author on reasonable request.
